# The Nation's Fund for Nurses

**Published:** 1921-02-26

**Authors:** Ethel Ayres Purdie

**Affiliations:** *Hon. Auditor, Certified Accountant*. Hampden House, 84 Kingsway, W.C. 2


### The Nation's Fund for Nurses.

*Statement of Receipts and Payments for the period June 6, 1917, to December 31, 1919.*

| Receipts. | £ | s. | d. |
|---|---|---|---|
| To Donations ... | 12,759 | 5 | 0 |
| ,, Collections ... | 4,522 | 12 | 4 |
| ,, Collecting Boxes, Cards and Books ... | 68 | 12 | 9 |
| ,, Schools and Colleges ... | 100 | 10 | 10 |
| ,, Entertainments ... | 33,505 | 8 | 1 |
| ,, Sale of Goods ... | 891 | 11 | 1 |
| ,, Rent (sublet portion of premises) ... | 30 | 0 | 0 |
| ,, Tribute Fund ... | 78,401 | 0 | 7 |
| ,, College of Nursing, Limited, Endowment and Scholarship Fund ... | 18,636 | 2 | 9 |
| | £148,915 | 3 | 5 |

| Payments. | £ | s. | d. | £ | s. | d. |
|---|---|---|---|---|---|---|
| By Advertisements and Press ... | | | | 4,214 | 18 | 5 |
| ,, Printing and Stationery ... | | | | 982 | 9 | 8 |
| ,, Salaries and Bonus ... | | | | 1,192 | 10 | 0 |
| ,, Office Expenses ... | | | | 474 | 0 | 0 |
| ,, Insurance ... | | | | 25 | 15 | 9 |
| ,, Bank Charges ... | | | | 7 | 19 | 9 |
| ,, Petty Cash ... | | | | 161 | 11 | 6 |
| ,, Purchase of Goods for Sale ... | | | | 22 | 14 | 0 |
| ,, Boxes and Books for Collections ... | | | | 39 | 7 | 6 |
| ,, Entertainments ... | | | | 966 | 13 | 0 |
| | | | | 8,087 | 19 | 7 |
| ,, Tribute Fund— | | | | | | |
| Grants to Nurses in Distress ... | 2,144 | 11 | 6 | | | |
| Returned to Ireland for Local Administration ... | 10,037 | 3 | 9 | | | |
| | | | | 12,181 | 15 | 3 |
| ,, College of Nursing, Limited— | | | | | | |
| For Endowment ... | 35,650 | 0 | 0 | | | |
| For Scholarships ... | 556 | 9 | 0 | | | |
| | | | | 36,206 | 9 | 0 |
| ,, London Centre, College of Nursing, Ltd. ... | | | | 3,000 | 0 | 0 |
| | | | | 59,476 | 3 | 10 |
| ,, Petty Cash in Hand ... | | | | 8 | 7 | 6 |
| ,, Cash at Bank—Current Account ... | | | | 7,248 | 3 | 5 |
| ,, ,, ,, Deposit ,, ... | | | | 5,350 | 0 | 0 |
| ,, 4 per cent. Funding Loan ... | | | | 6,800 | 0 | 0 |
| ,, National War Bonds ... | | | | 65 | 0 | 0 |
| ,, Vested in Trustees— | | | | | | |
| British Red Cross Society Grant to Tribute Fund | | | | 50,000 | 0 | 0 |
| Women's Emergency Corps Grant to Tribute Fund for Pensions for Nurses ... | | | | 2,767 | 8 | 8 |
| ,, 4 per cent. Funding Loan (Tribute Fund) ... | | | | 17,200 | 0 | 0 |
| | | | | £148,915 | 3 | 5 |

I have audited the above Statement of Receipts and Payments with the Books, Accounts, and Vouchers, and certify it to be correct. I have verified the Cash at Bank and the Securities, including those held in Trust by the Charity Commissioners.

Hampden House, 84 Kingsway, W.C. 2.

*December* 20, 1920.

ETHEL AYRES PURDIE, F.L.A.A., *Hon. Auditor,*

*Certified Accountant.*

